# Corpse or not? Two peculiar cases of misidentification

**DOI:** 10.1007/s12024-024-00799-3

**Published:** 2024-03-15

**Authors:** S. Plenzig, J. Helmus, M. Weber, M. A. Verhoff, V. Hachmann

**Affiliations:** 1 Institute of Legal Medicine, University Hospital Frankfurt, Goethe University, Kennedyallee 104, 60596 Frankfurt am Main, Germany; 2 University Hospital Halle (Saale), Institute of Legal Medicine, Franzosenweg 1, 06112 Halle (Saale), Germany; 3 Institute of Legal Medicine, Sana Kliniken Duisburg GmbH, Zu den Rehwiesen 9-11, 47055 Duisburg, Germany

**Keywords:** Misidentification, Primary external postmortem examination, Sex doll, Autopsy

## Abstract

Irrespective of whether they are intended for collectors or for the fetish market, dolls are being produced to look more and more realistic with such a degree of life-like detail that they can be mistaken for a real person. This paper reports two cases of misidentification due to this increasing similarity: In the first case, a sex doll was mistaken for a corpse; in the second case, a corpse was mistaken for a doll. While in the latter case, only medical laypersons were at the discovery site, in the first case, an emergency doctor had issued a medical certificate of death for the purported corpse. The medicolegal examiner who was subsequently called to the scene could still rectify the misconception on-site. Mistakes of this nature are likely rare phenomena. It, however, remains to be seen if the increasingly life-like appearance of dolls on the one hand, and the increasingly doll-like appearance of some people, e.g., through cosmetic surgery, will lead to a rise in such cases. To avoid misidentification as in the first reported case, it is essential to prepare medical students well for the task of performing a primary external postmortem examination; it is equally important that fully-trained doctors regularly refresh their expertise in this respect.

## Introduction

Life-like dolls have become extremely popular in various circles, not least amongst collectors. Baby dolls, in particular, are frequently crafted in such realistic detail for this market segment that they may easily be mistaken for real babies [1]. Some models are even equipped with heartbeat and voice functions [1].

In addition to realistic infant simulacra, there exists a market for real-life-like adult dolls accessible through online platforms. However, these dolls are comparatively less prevalent as items sought for collection, and more commonly for fetishistic purposes [2, 3]. Termed as “sex dolls”, these entities may surpass 45 kg in weight and frequently command prices exceeding 1000 Euros [2, 3].

In the following, we report two cases related to the increasing similarity between dolls and humans: In the one case, a doll was mistaken for a corpse; in the other, a corpse was mistaken for a doll.

## Case report 1

In spring, the on-duty medicolegal examiner at the Institute of Legal Medicine in Halle (Saale), Germany, was called to the discovery site of a corpse by criminal police officers. Over the phone, a police officer explained that children playing on an unused lot had found a female corpse. The children’s parents had subsequently notified the police. According to the police officer, the corpse was already mummified. Furthermore, the emergency doctor who had been called to the scene had already verified death. Although he had touched the corpse, he had not disturbed its position in view of a possible crime setting. The doctor had also already issued a medical certificate of death for an unknown person.

When the medicolegal examiner arrived, the discovery site had been widely cordoned off. Numerous media representatives had also already arrived at the scene. In consultation with the police, it was agreed that the external postmortem examination would wait until the forensic investigation of the site had been concluded. Consequently, the medicolegal examiner initially limited their involvement to the visual inspection of the deceased individual.

**Fig. 1:**
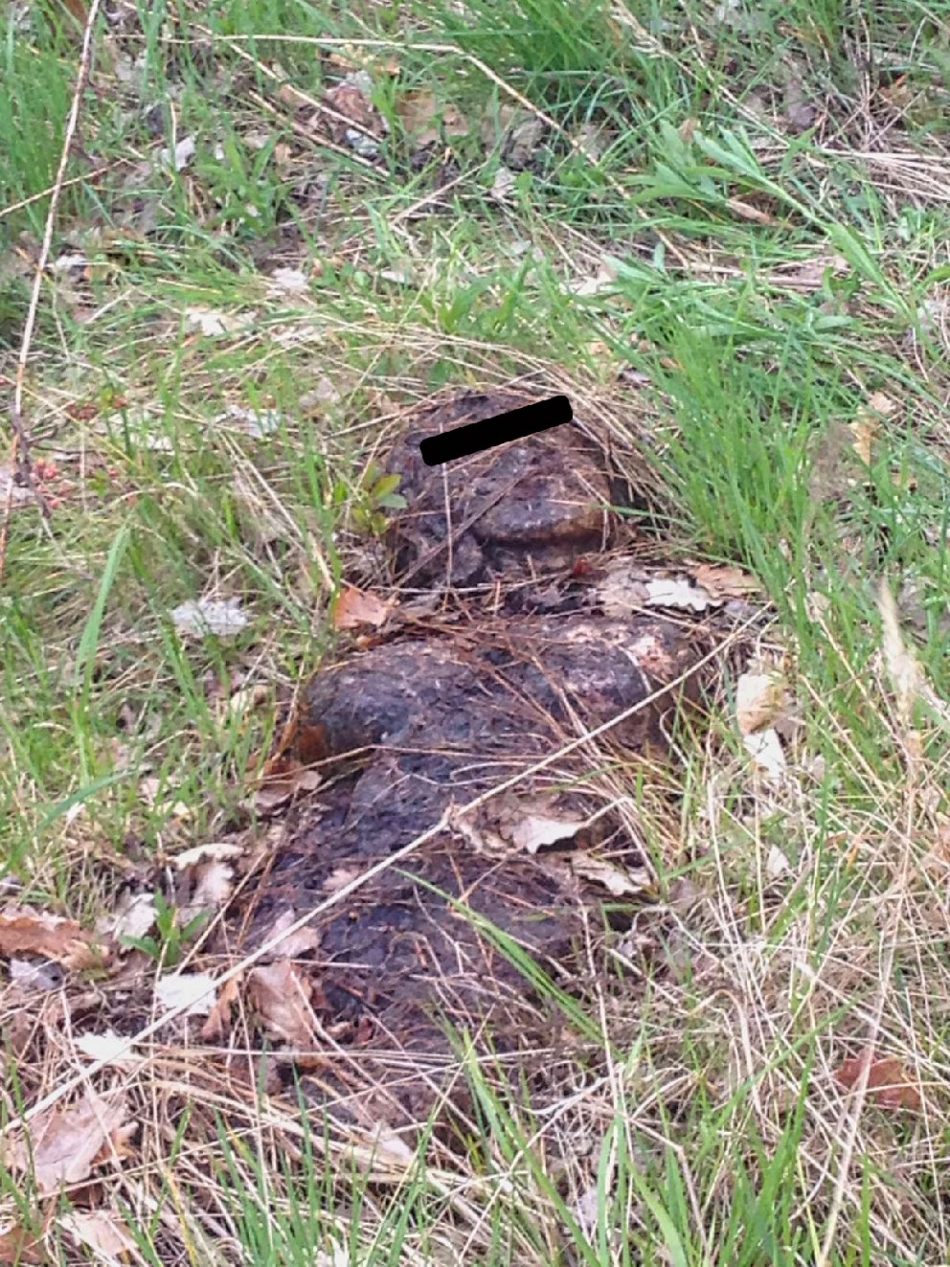
Overview of the discovery scene in Halle (Saale); Case report 1

The corpse lay in supine position on a densely overgrown, unused lot. The body was, to a large extent, covered with mud, grass, and leaves so that only the face and parts of the torso and thighs were exposed (Fig. 1).

**Fig. 2:**
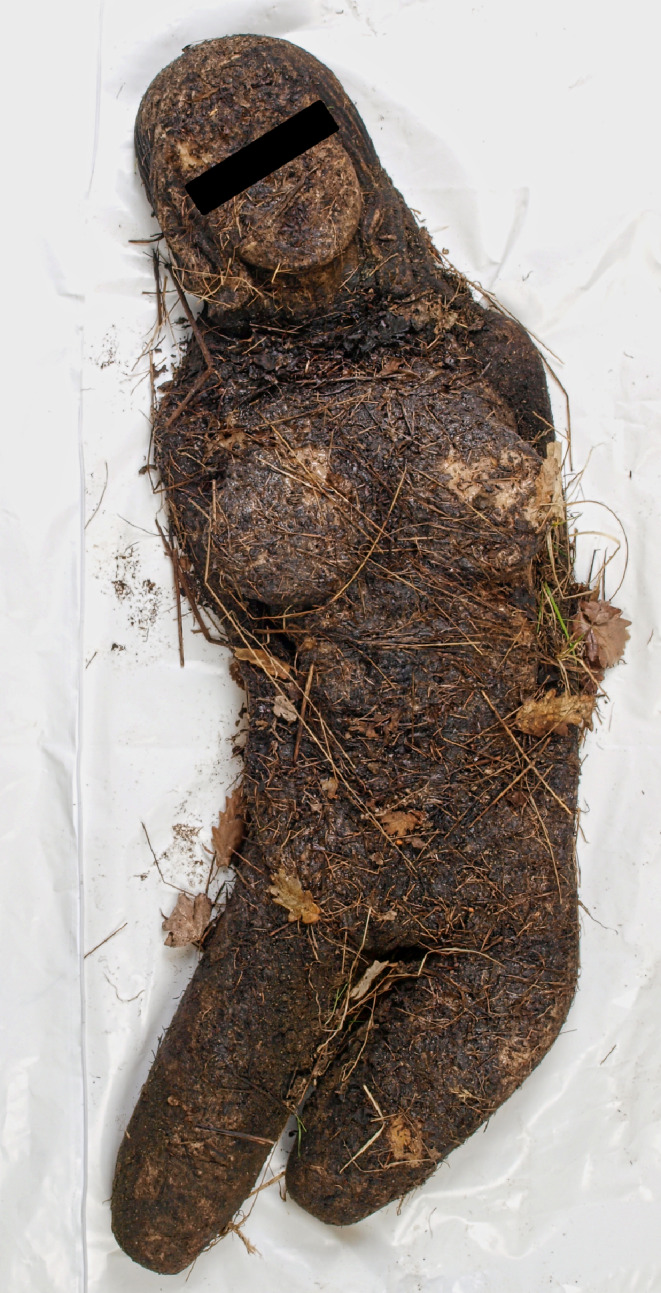
Recovered doll composed of considerably weathered silicon-like material; Case report 1

The conspicuously flattened facial structure of the deceased immediately drew attention upon initial observation. Closer inspection revealed that the right side of the torso was resting on an approximately fist-sized stone that had pushed the thorax inwards. After cautiously palpitating this area to see if broken ribs could be felt, the medicolegal examiner discovered that the presumed corpse was, in fact, a female body composed of silicon-like material (Fig. 2).

The medicolegal examiner immediately informed the responsible criminal police officers that the presumptive corpse was only a doll that had obviously lain on the site for some time and was therefore highly weathered.

After the doll had been recovered, it could be seen that it lacked lower legs and that the arms were continuous with the torso. This was initially not apparent because the body had largely been overgrown with grass.

The attending emergency physician subsequently annulled the provided death certificate and accompanying billing documentation. To put their minds to rest, the children who had discovered the “corpse” were informed that what they had found was a doll.

### Case report 2

During the winter season, a forced eviction from a private home within the jurisdiction of the Institute of Legal Medicine in Frankfurt am Main, Germany, was carried out.

**Fig. 3:**
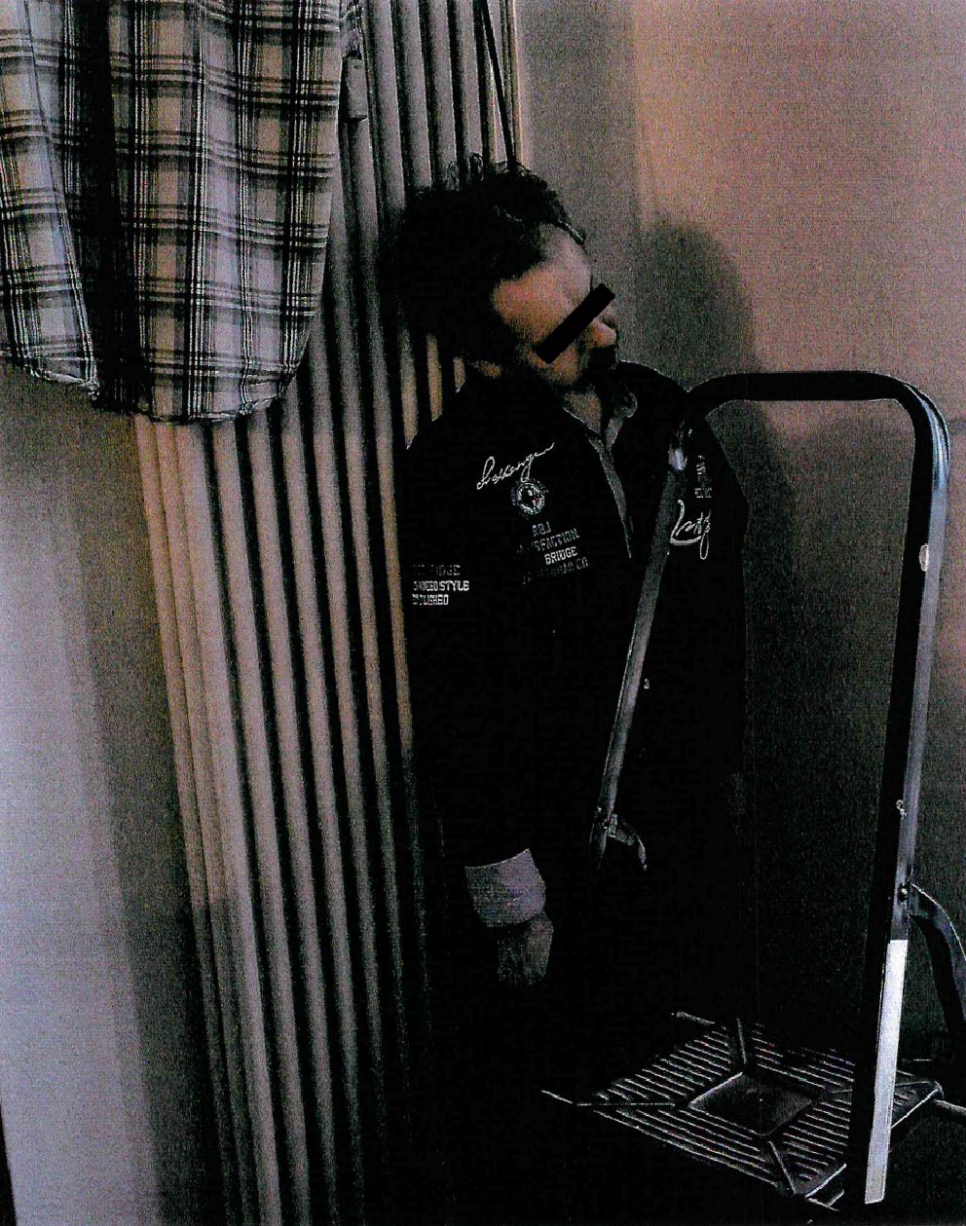
Overview of the discovery scene for the case from the Frankfurt catchment area, police photograph file; Case report 2

Co-workers from a removal company, the property manager, and a bailiff had appeared for the clearance. When the tenant failed to open the door to his home, they called for the superintendent, who had a master key to the apartment. Upon entering the apartment, they found a human, or human-like, body in front of a ceiling-height radiator. This body was attached to the upper section of the radiator by a cord around its neck (Fig. 3).

The superintendent explained to the others that the body was a life-like doll that he had seen draped around the apartment in various positions on numerous previous occasions. The items in the home were subsequently photographed and inventoried. In the inventory list, the “life-like doll” was listed as a “dummy” and assigned the inventory number 267. The cleared inventory, including the dummy, was put into storage in a large container on the premises of the removal company.

Approximately one month later, the bailiff instructed the removal company to dispose of the cleared inventory from the forced eviction, with the exception of the dummy, which was suited for auction because of its life-like appearance. The “doll” was subsequently removed from the container, transferred to a pallet, covered with fleece, and stored in one of the removal company’s storage halls.

A month later, the removal company’s manager contacted one of the bailiff’s colleagues to ask for guidance on how to further proceed with the dummy in storage because it had obviously begun to decompose. The decision was made to contact the police.

**Fig. 4:**
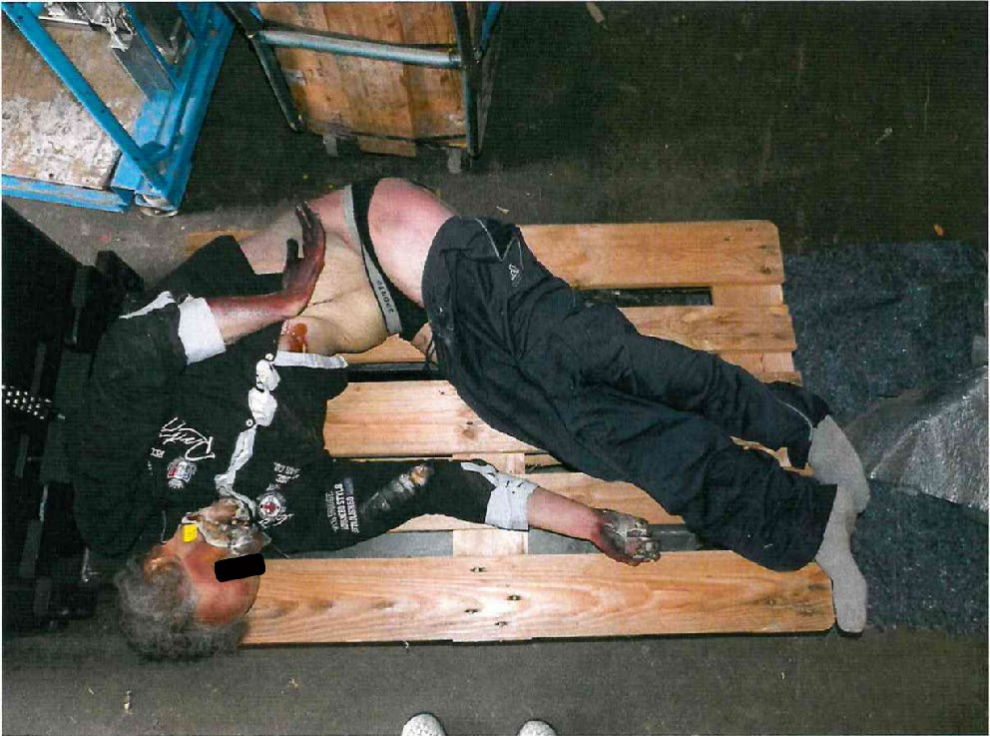
Scene found by the police on their arrival, police photograph file; Case report 2

Upon their arrival on the premises of the removal company, the police found the corpse of a man, in an advanced stage of putrefaction, on a pallet (Fig. 4).

The investigative authorities ordered a forensic autopsy, toxicological testing with alcohol concentration analysis, and DNA profiling.

The autopsy of the male corpse revealed the following findings: Apart from the advanced internal and external putrefactive changes and an inventory sticker bearing the number 267 on the corpse’s left cheek, cardiac hypertrophy, chronic obstructive pulmonary disease, and coronary artery disease were noted. There were no external or internal ligature marks. In as far as could be determined, there were no signs to suggest the man had been alive when he had ended up in the noose. The results of the toxicology tests and the alcohol analysis both did not indicate drug impairment or intoxication. Within the assessment constraints imposed by putrefaction, the only noteworthy histologic features to be found were pronounced myocardial fibrosis and, at best, early-stage liver cirrhosis. A comparative analysis of the DNA obtained from the deceased with that from a police databank allowed positive identification of the decedent as the tenant from the forced-eviction apartment.

During the ensuing police questioning of the people present at the original discovery site, the bailiff explained that the superintendent had been very convincing about the body being a life-like doll. In addition, the police records documented that the property manager also had stated that he had assumed that someone had wanted to play a “bad joke” on the persons tasked with the eviction, with the intention of giving them a fright. He had also added that the position of the body, had to him, appeared inconsistent with hanging because both of the body’s feet had stood firmly on the floor.

In the face of advanced putrefaction and the lack of findings consistent with hanging, the cause of death lastly remained undetermined. In view of these results and the inventory photographs that had captured the initial discovery scene in the apartment, there was no reason to exclude the possibility of death due to atypical hanging. Most notably, however, the investigation found no evidence of violence by another party.

## Discussion

According to the AWMF physician’s guidelines for the medical certification of death (*AWMF-Leitlinie Ärztliche Leichenschau*), the primary external postmortem examination and medical certification of death must be carried out with the greatest care. The same duties of diligence apply as for living patients [4]. The case from Halle (Saale) exemplifies the importance of this passage from the guidelines: If the emergency doctor had fully complied with this passage, a doll would likely not have been mistaken for a corpse. On the other hand, the circumstances of the “death” were unclear in this case and the discovery site was public. In the case of a real corpse, changes of the discovery site through a careful external postmortem examination might have impeded the collection of evidence and further investigation. In many German federal states, the “burial laws” in fact explicitly state that the primary external postmortem examination is to be broken off if there is any reason to suspect the possibility of a non-natural death [5, 6]. In the reported case, the setting in which the body was found could thus already be easily construed as such an indication. In addition, the weathered surface of the doll and the circumstance that it was partially overgrown with plants will significantly have contributed to the emergency doctor’s mistake, as illustrated in Fig. 1.

In contrast to the case from Halle (Saale), only medical laypersons–who likely had never, or only rarely, previously seen a corpse and hence will have had little, if any, expertise is this regard–were present at the discovery site of the case from Frankfurt. On the photograph of the fully clothed corpse, which was taken as part of the inventory process (Fig. 3), no late signs of death can be discerned. Accordingly, the corpse will not have exuded an unpleasant smell nor will obvious skin discolorations have been evident.

It is unknown whether and to what degree rigor mortis had set in when the body was placed in storage. A certain stiffness of the body may even have made the corpse appear more doll-like to the involved laypersons. In addition, it is likely that the co-workers of the removal company will have worn gloves when they moved the body into storage, so even if they had touched an area of naked skin, it is questionable if they would have been able to feel that the surface texture was inconsistent with that of a doll.

To avoid incidents like in the first case from Halle (Saale) in future–in particular in view of the incurred costs and the waste of time for the involved personnel–it is essential for all physicians who may be called in to certify death to be well trained to perform primary external postmortem examinations, and it is equally important that they regularly refresh their knowledge in this respect [4]. To have avoided a situation as in the second case from Frankfurt, the involved lay persons would have had to call in a doctor to perform a primary external postmortem examination. It is, however, debatable whether any doctor would have felt compelled to follow a request to ascertain whether a doll might not after all be a corpse. The only option would, therefore, have been to have notified the police as soon as the body had been discovered in the apartment.

Mistaking a doll for a dead person and a dead person for a doll are likely both equally rare phenomena. The two cases reported here illustrate that such misidentifications may, nonetheless, be encountered in medicolegal practice. On the one side, dolls are, for various reasons, being made to appear more and more realistic [1–3]; on the other side, there is also a trend towards special cosmetic surgery to make humans look doll-like [7]. It thus remains to be seen whether these two trends will lead to a future rise in misidentifications similar to those described in our report.

## Key points

1. Two remarkable cases of misidentification are presented.
2. The confusion of a doll with a corpse is probably a rare phenomenon.
3. It is essential for all physicians who may be called in to certify death to be well trained to perform primary external postmortem examinations.

## References

[1] https://www.reborntraumland.de/collections/bestsellers, last access 19.07.2022.

[2] https://www.realdoll24-shop.de/cat/index/sCategory/333, last access 19.07.2022.

[3] https://www.dollpark.com/sexpuppen, last access 11.06.2023.

[4] AWMF-Leitlinie Ärztliche Leichenschau; https://register.awmf.org/assets/guidelines/054-002l_S1_Regeln-zur-Durchfuehrung-der-aerztlichen-Leichenschau_2018-02_01.pdf, last access 28.09.2023.

[5] Gesetz über das Leichen-., Bestattungs- und Friedhofswesen des Landes Sachsen-Anhalt, § 6; https://www.landesrecht.sachsen-anhalt.de/bsst/document/jlr-BestattGSTpP6, last access 09.11.2023.

[6] Hessisches Friedhofs- und Bestattungsgesetz., § 11; https://www.rv.hessenrecht.hessen.de/bshe/document/jlr-BestattGHE2007rahmen/part/X, last access 09.11.2023.

[7] https://www.stern.de/kultur/-barbie---us-chirurg-bietet-schoenheits-ops-fuer-120-000-dollar-an-33687374.html, last access 28.09.2023.

